# Mutually Positive Regulatory Feedback Loop between Interferons and Estrogen Receptor-α in Mice: Implications for Sex Bias in Autoimmunity

**DOI:** 10.1371/journal.pone.0010868

**Published:** 2010-05-28

**Authors:** Ravichandran Panchanathan, Hui Shen, Xiang Zhang, Shuk-mei Ho, Divaker Choubey

**Affiliations:** 1 Department of Environmental Health, University of Cincinnati, Cincinnati, Ohio, United States of America; 2 Cincinnati Veterans Affairs Medical Center, Cincinnati, Ohio, United States of America; New York University, United States of America

## Abstract

**Background:**

Systemic lupus erythematosus (SLE), an autoimmune disease, predominantly affects women of childbearing age. Moreover, increased serum levels of interferon-α (IFN-α) are associated with the disease. Although, the female sex hormone estrogen (E2) is implicated in sex bias in SLE through up-regulation of IFN-γ expression, the molecular mechanisms remain unknown. Here we report that activation of IFN (α or γ)-signaling in immune cells up-regulates expression of estrogen receptor-α (ERα; encoded by the *Esr1* gene) and stimulates expression of target genes.

**Methodology/Principal Findings:**

We found that treatment of mouse splenic cells and mouse cell lines with IFN (α or γ) increased steady-state levels of ERα mRNA and protein. The increase in the ERα mRNA levels was primarily due to the transcriptional mechanisms and it was dependent upon the activation of signal transducer and activator of transcription-1 (STAT1) factor by IFN. Moreover, the IFN-treatment of cells also stimulated transcription of a reporter gene, expression of which was driven by the promoter region of the murine *Esr1* gene. Notably, splenic cells from pre-autoimmune lupus-prone (NZB × NZW)F_1_ female mice had relatively higher steady-state levels of mRNAs encoded by the IFN and ERα-responsive genes as compared to the age-matched males.

**Conclusions/Significance:**

Our observations identify a novel mutually positive regulatory feedback loop between IFNs and ERα in immune cells in mice and support the idea that activation of this regulatory loop contributes to sex bias in SLE.

## Introduction

Systemic lupus erythematosus (SLE) is a prototype autoimmune disease in which patients develop pathogenic autoantibodies against nuclear antigens and the disease involves multiple organs, including the kidneys [1], [2]. The disease has a strong sex bias and develops at a female-to-male ratio of 10∶1 [3]–[6]. The sex bias in SLE is thought to be influenced by sex hormones, such as estrogen and androgen [4]–[6]. Additionally, it has been noted [7] that ERα mRNA levels are significantly higher in peripheral blood mononuclear cells (PBMCs) from SLE patients as compared to normal controls. Moreover, the female sex hormone estrogen (E2) is known to have immunomodulatory effects [5]. For example, in vitro treatment of PBMCs from SLE patients with estrogen results in polyclonal activation, secretion of antibodies to double-stranded DNA, and defects in apoptosis of immune cells [5], [6].

Sex hormones also influence the pathogenesis of murine lupus [8]–[11]. For example, in (NZB × NZW) F_1_ mouse model of SLE disease, female mice develop the disease earlier and have shorter life spans than males [8]. In contrast, castrated male (NZB × NZW) F_1_ mice have earlier onset of lupus and shorter life span than their intact littermates [9]. In addition treatment with estrogen exacerbates disease activity and causes early mortality [10], [11].

Estrogen functions by activating one of its two nuclear receptors, ERα and ERβ [12], [13]. Both receptors are expressed in most immune cells [14]. Several recent studies involving mouse models of SLE disease have provided evidence for a prominent role of ERα in the development of lupus disease [10], [11], [15], [16]. Interestingly, the ERα deficiency in (NZB × NZW) F1 female mice attenuated glomerulonephritis and increased survival of mice [15]. Of note, the increased survival of ERα deficient female mice was associated with reduced development of anti-chromatin and anti-dsDNA antibodies as well as reduced serum levels of IFN-γ [15]. Moreover, E2 is known to promote IFN-γ production by invariant natural killer T cells [17], dendtritic cells [18], and splenocytes [19]. Interestingly, the participation of IFN-γ in lupus pathogenesis has been demonstrated in mice [20] and in SLE patients [21]. Consistent with a role for IFN-γ in the development of lupus disease, deletion of the IFN-γ receptor [22] or depletion of IFN-γ in lupus-prone (NZB × NZW)F_1_ mice [23] prevents autoantibody production and glomerulonephritis. These observations have demonstrated a role for both estrogen and IFN-γ signaling in the development of lupus disease in mouse models.

Studies have indicated that SLE patients with active disease have elevated serum levels of type I IFNs (IFN-α/β) [20], [24]. It has been proposed that tissue damage, either as a result of infections or sterile injuries could be source of apoptotic debris and, thus, autoantigen, which in turn can induce the type I IFN production [25]. Moreover, consistent with increased serum levels of IFN-α in SLE patients, PBMCs from SLE patients also exhibit a gene expression profile indicative of an active IFN-α signaling [24], [25]. The role of type I IFN-signaling has also been investigated in mouse models of SLE [20]. It is known that mice that are deficient in the type I receptor do not develop the disease [20]. Interestingly, a comparison of gene expression analysis between pre-autoimmune (NZB × NZW) F_1_ and MRL/*lpr* mice has suggested that mononuclear cells from (NZB × NZW) F_1_ female mice express higher levels of IFN-α and IFN-γ-inducible genes than the MRL/*lpr* mice [26]. Moreover, our work revealed that the type I interferon receptor deficiency reduces lupus-like disease in the lupus-prone NZB mice [27]. Although, the above studies using mouse models of SLE and human SLE patients have also provided evidence for a role for IFN-signaling in lupus disease, it remains unclear whether the increased levels of IFNs contribute to sex bias in SLE.

Type I IFNs are multifunctional cytokines with potent immunomodulatory activities [20]. In IFN-responsive cells, binding of Type I IFNs to cell surface receptor results in activation of the receptor-associated Janus tyrosine kinases, Jak1 and Tyk2, which in turn leads to tyrosine phosphorylation and activation of latent transcription factors termed STATs [28]. The activated STATs then form homodimers or heterodimers and translocate into the nucleus, bind to conserved promoter sequences termed interferon stimulated response element (ISRE), and induce the transcription of IFN-responsive genes. The IFN-stimulated gene factor 3 (ISGF3), which includes IRF9, and Stat1:Stat2 heterodimers binds to the ISRE sequence and activates transcription of the target genes. Notably, the type IFNs can also activate transcription of certain IFN-responsive genes independent of the Jak/STAT pathway [29].

Numerous studies have suggested role for IFN [20], [24], [25] and estrogen [4]–[6] signaling in the development of SLE. Moreover, the female hormone estrogen is known to up-regulate the expression of IFN-γ in immune cells [15], [17]–[19]. Therefore, we explored whether IFNs could regulate expression of ERα. Here, we report that the IFNs (α or γ) up-regulate the expression of ERα and stimulate the ERα-mediated transcriptional activation of genes.

## Materials and Methods

### Mice and Cells

All mice were handled in accordance with good animal practice as defined by the requirement of the National Institutes of Health and the University of Cincinnati's animal committee, and all experimental protocols that are used in this manuscript were approved (approval #07-05-24-01) by the University of Cincinnati's Animal Care and Use Committee. Age-matched (∼6–8 weeks old) male and female C57BL/6J and (NZB × NZW) F1 mice were purchased from The Jackson Laboratory. Age-matched wild type and homozygous Stat1-null 129S6/SvEv-Stat1tm1Rds mice (age ∼6–8 weeks) [30] were purchased from Taconic Farm (Hudson, NY). All mice were housed in a germ-free Laboratory Animal and Medical Services facility of the University of Cincinnati.

### Splenocyte Isolation, Cell culture, and Hormone Treatment

Total single cell splenocytes were prepared from male or female mice as described previously [31]. Unless, otherwise indicated, splenic cells from two or more age-matched male or female mice were pooled to prepare total RNA or protein extracts. Splenic B or T cells were purified from total splenic cells using magnetic beads from Miltenyi Biotech (Auburn, CA) as described previously [31]. Estrogen-responsive mouse breast cancer cell line WT276 [31] was generously provided by Dr. JoEllen Welsh, University of Notre Dame, Notre Dame, IN. Mouse RAW264.7 macrophage cell line was purchased from ATCC. Cells were maintained in DMEM medium supplemented with 10% fetal bovine serum and 1× antibiotic-antimycotic solution (Invitrogen, Carlsbad, CA). When indicated, mouse splenic cells or mouse cell lines were treated with either IFN-α (1,000 u/ml; Universal IFN-α, from R & D Systems, Minneapolis, MN) or murine IFN-γ (10 ng/ml) for the indicated duration. For treatment of mouse splenocytes or cell lines with 17-β-estradiol (E2; 1–10 nM), cells were cultured in phenol red-free RPMI 1640 medium (Invitrogen) and the medium was supplemented with 10% charcoal-stripped fetal bovine serum (Invitrogen). Splenocytes (5-8×10^6^ cells) were used to isolate total RNA using TRIzol (Invitrogen).

### Plasmids

The ERE-luc-reporter plasmid has been described previously [32]. The ISRE-luc-reporter plasmid was purchased from B D Biosciences (San Jose, CA). A plasmid reporter construct in which the murine *Esr1* gene promoter-region (∼5-kb) is linked to the β-galactosidase reporter gene was generously provided by Dr. Alessandro Weisz (Seconda Università degli Studi di Napoli, Italy) and the plasmid construct has been described [33].

### Reporter Assays

For reporter assays, sub-confluent cultures of WT276 cells (in 6-well plates) were transfected with the indicated reporter plasmids (either ERE-luc or ISRE-luc; 1.8 µg plasmid DNA) and a second reporter plasmid pRL-TK (0.2 µg;), as an internal control to normalize the transfections efficiency, using the FuGENE 6 transfection reagent (Roche, Indianapolis, IN), as suggested by the supplier. When indicated, cells were either treated with ethanol (vehicle), the indicated concentration of E2, or IFN-α (1,000 u/ml) for 18 h. Unless, otherwise indicated, cells were harvested between 40 and 45 h after transfections. Cells were lysed, and the firefly and *Renilla* dual luciferase activities were determined using a dual luciferase assay kit (Promega, Madison, WI) as described previously [31]. For β-galactosidase assay, a Galacto-Light Plus Systems kit (Applied Biosystems, Bedford, MA) was used following the manufacturer's instructions. For this assay, the units were normalized for total protein content measured with the Bio-Rad protein assay reagent.

### RT-PCR and Quantitative Real-Time PCR Analysis

Splenocytes (5-8×10^6^ cells) or purified (93–95% pure) splenic B or T cells (2-3×10^6^ cells) were used to isolate total RNA using TRIzol (Invitrogen). Total RNA (2.0 µg) was used for or RT-PCR reaction. We used the Superscript one-step RT-PCR system from Invitrogen. Primers for the murine *Esr1* gene that were used (forward: 5′-aattctgacaatcgacgccag- 3′; backward: 5′-gtgcttcaacattctccctcctc-3′) gave a single band of 345 base pair. Quantitative real-time TaqMan PCR technology (Applied Bio-systems, Foster City, CA, USA) was used to quantitate the steady-state levels of mRNAs. The PCR cycling program consisted of denaturation at 95°C for 10 min, 40 cycles at 95°C for 15 seconds, followed by annealing and elongation at 60°C for 1 min. The TaqMan assays for *Serpinb2* (Assay Id*#* Mm00440905_m1), *Rab10* (#Mm00489481_m1), *Ifi202* (#Mm03048198 _m1), *Mx1* (#Mm00487796_m1), *Syn25A* (#Mm00836412_m1), *Esr1* (Assay Id#Mm00433 149_m1), and the endogenous control β*2-microglobulin* (Assay Id#Mm00437762_ m1) were purchased from Applied Bio-systems (Foster City, CA) and used as suggested by the supplier.

### Immunoblot Analysis

Total splenocytes, purified splenic B or T cells, WT276, or RAW264.7 cells were collected in PBS and re-suspended in a modified radio-immune precipitation assay (RIPA) lysis buffer (50 mM Tris-HCl, pH 8.0, 250 mM NaCl, 1% Nonidet P-40, 0.5% sodium deoxycholate, 0.1% SDS), supplemented with protease inhibitors (Roche Diagnostics, Mannheim, Germany) and phosphatase inhibitors (Sigma) and incubated at 4°C for 30 min. Cell lysates were sonicated briefly before centrifugation at 14,000 rpm in a microcentrifuge for 10 min at 4°C. The supernatants were collected, and the protein concentration was measured by Bio-Rad protein assay kit. Equal amounts of protein were processed for immunoblotting. Antibodies to detect mouse ERα (sc-542; MC-20), c-Jun (sc-1694; H-79), p-c-Jun (sc-822; KM-1), and β2-mcroglobulin (sc-13565) were purchased from Santa Cruz Biotechnology (Santa Cruz, CA). Antibodies to detect STAT1 (#9172), p-STAT1 (#9177), and β-actin (#4967) were purchased from Cell Signaling Technology (Danvers, MA).

### Statistical Analysis

Data are presented as the means ± S.E. For statistical comparisons between two groups, Student's two-tailed *t* test was used. *p*<0.05 was considered significant.

## Results

### IFN-Treatment Increases Steady-state Levels of ERα mRNA and Protein

**Figure 1:**
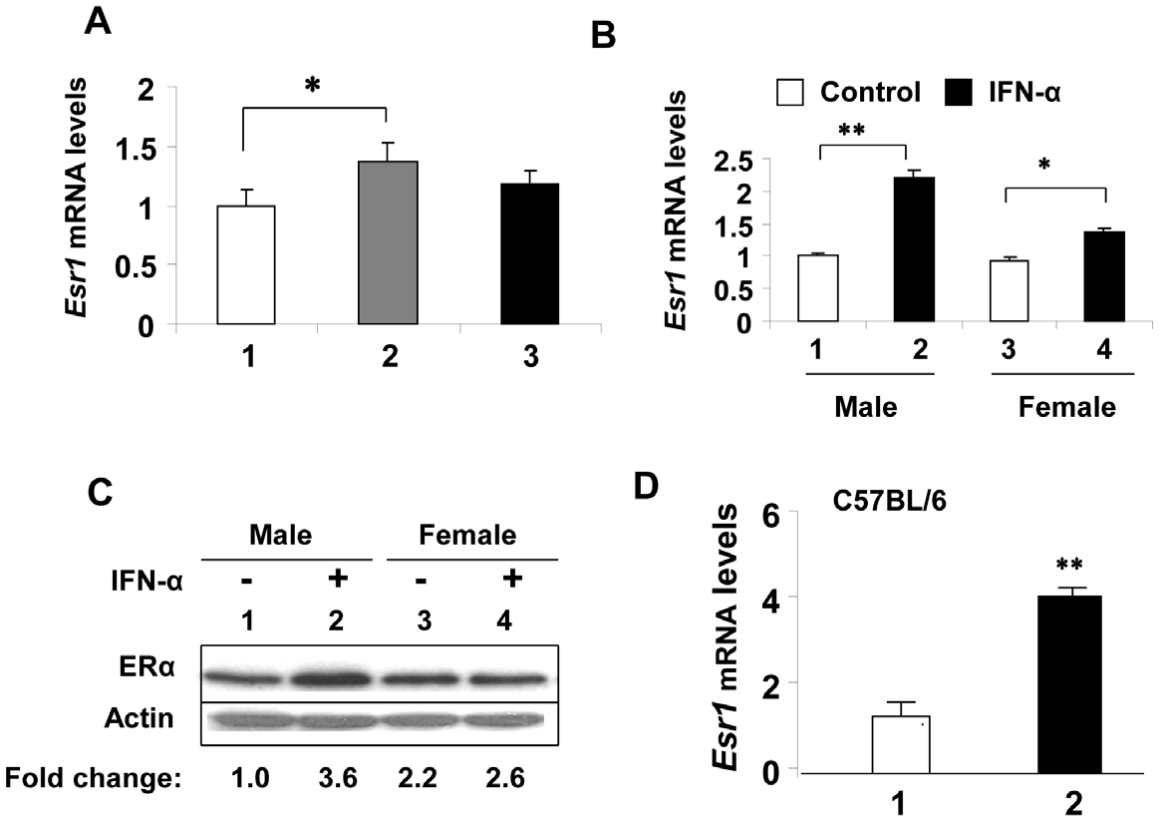
IFN-treatment increases steady-state levels of ERα mRNA and protein in C57BL/6 splenic cells. (A) Total RNA was isolated from control (column 1), IFN-α (column 2), or IFN-γ (column 3) treated total splenic cells that were prepared from female (age ∼8 weeks) C57BL/6 mice. The RNA was analyzed for steady-sate levels of *Esr1* mRNA by quantitative real-time PCR. The ratio of the *Esr1* mRNA to β2-microglobulin mRNA was calculated in units (one unit being the ratio of *Esr1* mRNA to β2-microglobulin mRNA). Results are mean values of triplicate experiments and error bars represent standard deviation (^*^ *p*<0.05). (B) Total RNA was isolated from control (column 1 and 3) or IFN-α (column 2 and 4) treated splenic cells that were prepared from either male (age ∼8 weeks) or age-matched female C57BL/6 mice. The RNA was analyzed by quantitative real-time PCR for the steady-sate levels of Esr1 mRNA as described in (A). Results are mean values of triplicate experiments and error bars represent standard deviation (^*^ *p*<0.05; ^**^ *p*<0.005). (C) Total protein extracts were prepared from control (lanes 1 and 3) or IFN-α (lanes 2 and 4) treated splenic cells that were isolated from either male (age ∼8 weeks) or age-matched female C57BL/6 mice. The total cell extracts were analyzed by immunoblotting using antibodies specific to the indicated proteins. Fold change in ERα protein levels is indicated below the Figure. (D) Total RNA was isolated from purified splenic T cells (column 1) or B cells (column 2) isolated from female (age ∼8 weeks) C57BL/6 mice. The RNA was analyzed by quantitative real-time PCR for steady-sate levels of *Esr1* mRNA. Results are mean values of triplicate experiments and error bars represent the standard deviation (^**^ *p*<0.005).

To explore whether IFNs could regulate the expression of ERα, we treated total splenic cells from non lupus-prone C57BL/6 female mice with either the murine IFN-α (1,000 u/ml) or IFN-γ (10 ng/ml) and compared steady-state levels of ERα mRNA by quantitative real-time PCR. As shown in Fig. 1A, treatment of cells with IFN-α or IFN-γ measurably increased the steady-state levels of ERα mRNA. Interestingly, the increase was more pronounced (∼50% versus 30%) after the IFN-α than IFN-γ treatment. Next, we compared the IFN-mediated increase in ERα mRNA levels between C57BL/6 male and age-matched female mice. As shown in Fig. 1B, treatment of total splenic cells with IFN-α measurably increased the steady-state levels of ERα mRNA (Fig 1B) and protein (Fig. 1C) in both male and female mice. Notably, the extent of IFN-α-mediated increase in ERα mRNA (Fig. 1B) and protein (Fig. 1C) was more appreciable (∼2-4-fold) in male splenic cells than the age-matched females. Moreover, basal levels of ERα protein were reproducibly ∼2-fold higher in splenic cells from females than the age-matched males in several experiments. Because we recently reported that the murine splenic B cells express relatively higher levels of ERα than T cells [31], we also compared ERα mRNA levels between purified splenic T and B cells. As shown in Fig. 1D, as compared to T cells, the B cells had significantly (∼4-fold) higher levels of ERα mRNA. Together, these observations revealed that IFN-α or γ treatment of C57BL/6 splenic cells increases steady-state levels of ERα mRNA and protein.

**Figure 2:**
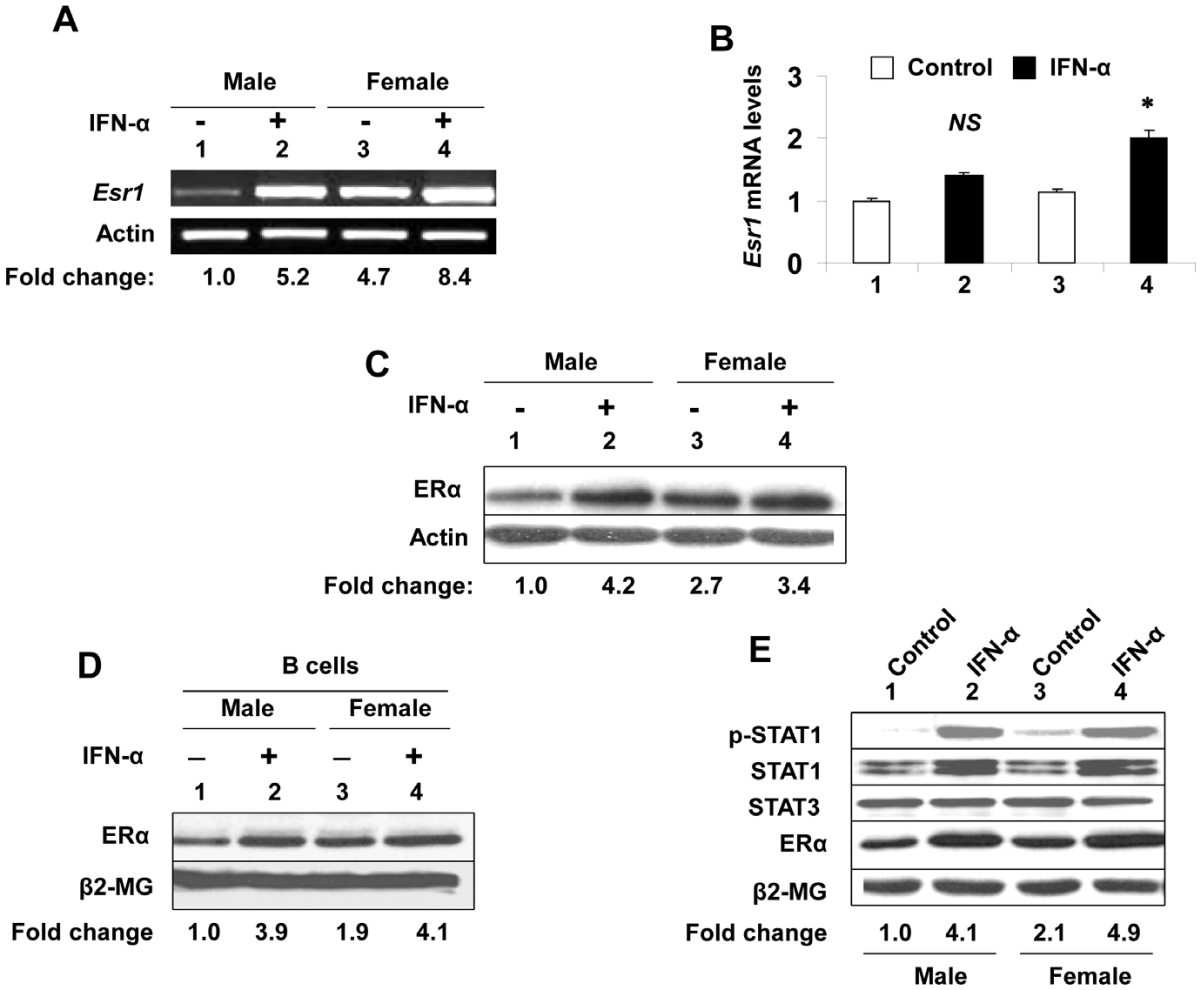
IFN-treatment increases steady-state levels of ERα mRNA and protein in splenic cells from lupus-prone (NZB × NZW) F_1_ mice. (A) Total RNA isolated from control (lanes 1 and 3) or IFN-α (lanes 2 and 4) treated total splenic cells that were isolated from either male (age ∼8 weeks) or age-matched female (NZB × NZW) F_1_ mice. The total RNA was analyzed for steady-state levels of *Esr1* mRNA by semi-quantitative RT-PCR. Fold change in *Esr1* mRNA levels is indicated below the Figure. (B) Total RNA isolated from control (columns 1 and 3) or IFN-α (columns 2 and 4) treated total splenic cells that were isolated from either male (age ∼8 weeks) or age-matched female (NZB × NZW) F_1_ mice. The total RNA was analyzed for the steady-state levels of *Esr1* mRNA by quantitative real-time PCR. Results are mean values of triplicate experiments and error bars represent standard deviation (^*^ *p*<0.05; NS, not significant). (C) Total protein extracts were prepared from control (lanes 1 and 3) or IFN-α (lanes 2 and 4) treated splenic cells that were isolated from either male (age ∼8 weeks) or age-matched female (NZB × NZW)F_1_ mice. The total cell extracts were analyzed by immunoblotting using antibodies specific to the indicated proteins. Fold change in ERα protein levels is indicated below the Figure. (D and E) Total protein extracts were prepared from control (lanes 1 and 3) or IFN-α (lanes 2 and 4) treated purified splenic B cells that were isolated from either male (age ∼8 weeks) or age-matched female (NZB × NZW) F_1_ mice. The total cell extracts were analyzed by immunoblotting using antibodies specific to the indicated proteins. Fold change in ERα protein levels is indicated below the Figures.

We also tested whether IFN-treatment of splenic cells from pre-autoimmune (age ∼8-weeks) lupus-prone (NZB × NZW) F_1_ mice also increases ERα expression. As shown in Fig. 2, the IFN-α treatment of splenic cells from both male and female mice increased the steady-state levels of ERα mRNA as determined by both semi-quantitative (Fig. 2A) and quantitative real-time PCR (Fig. 2B). Consistent with these observations, IFN-α treatment of splenic cells also increased ERα protein levels ∼2-4-fold (Fig. 2C). Interestingly, basal levels of ERα mRNA (Fig. 2A, compare lane 3 with 1) and protein (Fig. 2C, compare lane 3 with 1) were measurably higher in (NZB × NZW) F_1_ female mice as compared to the age-matched male mice. Furthermore, consistent with our above and the previous [31] observations, the basal levels of ERα protein were about two-fold higher in purified splenic B cells from female mice than the age-matched male mice and IFN-α treatment of B cells further increased the levels of ERα protein ∼2-4-fold (Fig. 2D).

Binding of type I IFNs to the cell surface receptor activates multiple signaling pathways, including the classical Jak/STAT pathway, which lead to the transcriptional activation of the IFN-inducible genes [28], [29]. Therefore, we explored whether the IFN-treatment of splenic B cells activates the STAT1 transcription factor. As shown in Fig. 2E, treatment of splenic B cells from (NZB × NZW) F_1_ mice with IFN-α increased the activating phosphorylation of STAT1 and basal levels of STAT1 protein in both male and female splenic cells. Again, the extent of IFN-α-mediated increase in the phosphorylation of STAT1 and increase in the levels of ERα protein were more appreciable in male B cells than the age-matched females. Moreover, the basal levels of phospho-STAT1 and ERα protein were reproducibly higher in splenic cells from females than the age-matched males. Together, these observations suggested that the basal levels of ERα mRNA and protein are relatively higher in splenic cells from lupus-prone (NZB × NZW) F_1_ females than the age and strain-matched males and IFN-α (or IFN-γ) treatment of splenic cells increases the steady-state levels of ERα mRNA and protein in both males and females. Additionally, these observations suggested that the basal levels of phospho-STAT1 and ERα in female B cells were relatively higher than the age-matched males and the IFN-α treatment of B cells increased the steady-state levels of both phospho-STAT1 and ERα further.

**Figure 3:**
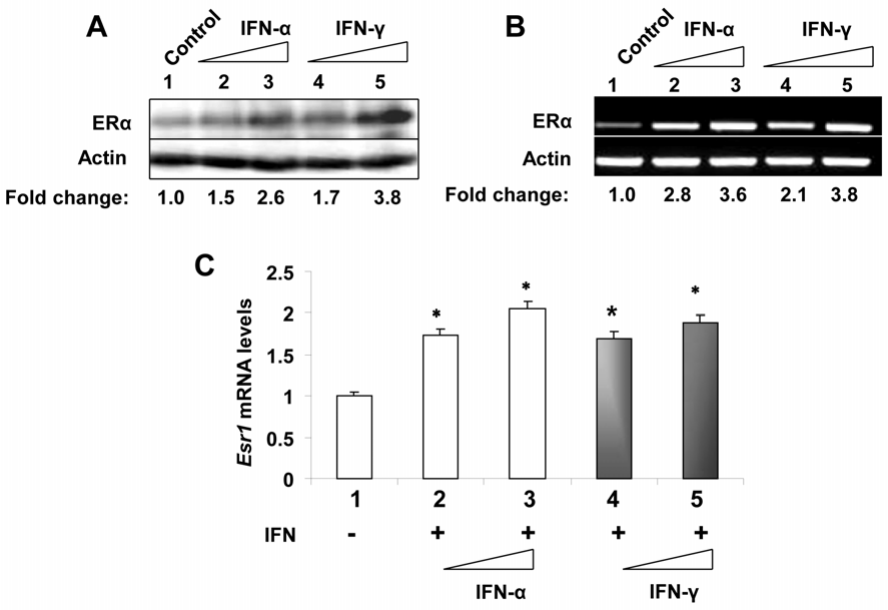
IFN-treatment increases steady-state levels of ERα mRNA and protein in mouse breast cancer cell line WT276. (A) Total protein extracts were prepared from control (lane 1), increasing concentrations (1,000 or 2000 u/ml) of IFN-α (lanes 2 and 3, respectively) or IFN-γ (5 or 10 ng/ml; lanes 4 and 5, respectively) treated WT276 cells. As a negative control, we also included extracts from AKR-2B cells. The extracts were analyzed by immunoblotting using the antibodies specific to the indicated proteins. Fold change in ERα protein levels is indicated below the Figure. (B) Total RNA was isolated from control (lane 1), increasing concentrations of IFN-α (lanes 2 and 3) or IFN-γ (lanes 4 and 5) treated WT276 cells. As a positive control, we also included RNA from splenic cells. The total RNA was analyzed for steady-state levels of *Esr1* mRNA by semi-quantitative RT-PCR. Fold change in ERα mRNA levels is indicated below the Figure. (C) Total RNA was isolated from control (column 1), increasing concentrations of IFN-α (columns 2 and 3) or IFN-γ (columns 4 and 5) treated WT276 cells. The total RNA was analyzed for steady-state levels of *Esr1* mRNA by quantitative real-time PCR. Results are mean values of triplicate experiments and error bars represent standard deviation (^*^ *p*<0.05).

To investigate the molecular mechanisms by which IFN-signaling increases the steady-state levels of ERα mRNA and protein in mouse splenic cells, we investigated the effect of IFN-treatment of WT276 mouse breast cancer cell line (an ERα-positive cell line; ref. 31) on steady-state levels of ERα mRNA and protein. As shown in Fig. 3A, treatment of cells with either IFN-α or IFN-γ increased the ERα protein levels and the extent of the increase was dependent on the dose of IFN-α or IFN-γ. Moreover, steady-state levels of ERα mRNA were also increased after IFN-α or IFN-γ treatment as determined by semi-quantitative RT-PCR (Fig. 3B) and quantitative real-time PCR (Fig. 3C). Similarly, treatment of mouse macrophage cell line RAW264.7 with IFN-α or IFN-γ also increased steady-state levels of ERα mRNA and protein in a dose-dependent manner (data not shown). Together, these observations revealed that IFN-α or IFN-γ treatment of mouse cell lines that express ERα also increased the steady-state levels of ERα mRNA and protein.

### Interferon-signaling Increases ERα mRNA Levels Primarily by Transcriptional Mechanism

**Figure 4:**
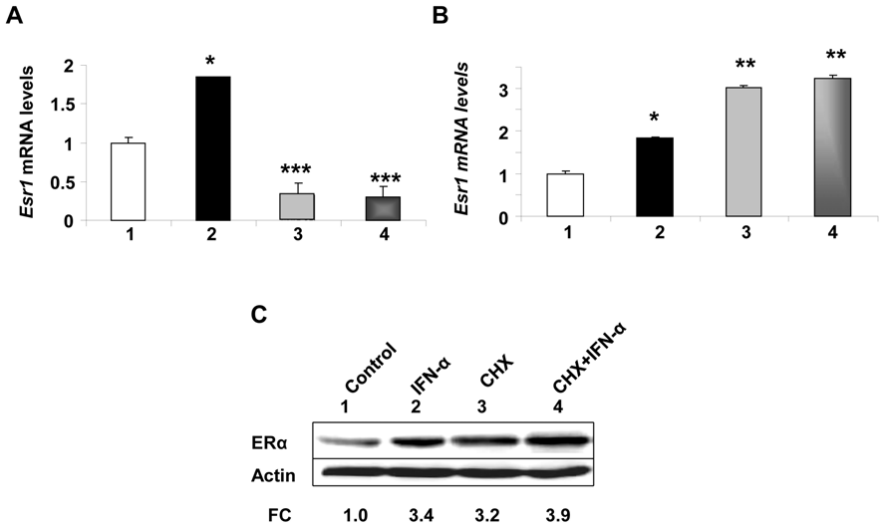
Interferon-signaling increases ERα mRNA levels primarily by Transcriptional mechanism. (A) Total RNA was isolated from control (column 1), IFN-α (column 2), actinomycin D (column 3), or both IFN-α and actinomycin D (column 4) treated WT276 cells. The RNA was analyzed by quantitative real-time PCR for steady-sate levels of *Esr1* mRNA. Results are mean values of triplicate experiments and error bars represent standard deviation (^*^ *p*<0.05; ^***^ *p*<0.0005). (B) Total RNA was isolated from control (column 1), IFN-α (column 2), cycloheximide (column 3), or both IFN-α and cycloheximide (column 4) treated WT276 cells. The RNA was analyzed by quantitative real-time PCR for steady-sate levels of Esr1 mRNA. Results are mean values of triplicate experiments and error bars represent standard deviation (^*^ *p*<0.05; ^**^ *p*<0.005). (C) Total cell extracts were prepared from control (lane 1), IFN-α (lane 2), cycloheximide (lane 3), or both IFN-α and cycloheximide (lane 4) treated WT276 cells. The cell extracts were analyzed by immunoblotting using antibodies specific to the indicated proteins. Fold change in ERα protein levels is indicated below the Figure.

Regulation of steady-state levels of ERα mRNA and protein is complex and the regulation may depend on the cell type [10]–[13]. Moreover, the promoter of the murine *Esr1* gene is reported to be relatively weak and does not contain a TATA box [32], [33], [34]. Therefore, to investigate the molecular mechanisms by which IFN-α treatment of cells increased the expression of ERα, we compared levels of ERα mRNA in WT276 cells that were treated with IFN-α alone or along with actinomycin-D, an inhibitor of gene transcription [35]. As shown in Fig. 4A, treatment of cells with the inhibitor alone decreased basal steady-state levels of ERα mRNA about 60% (compare column 3 with 1). Interestingly, co-treatment of cells with IFN-α plus the inhibitor abrogated the IFN-α-mediated increase in the ERα mRNA levels (compare column 4 with 2). Moreover, treatment of WT276 cells with IFN-α, cycloheximide (an inhibitor of protein synthesis) or both IFN-α plus cycloheximide increased steady-state levels of ERα mRNA (Fig. 4B) and protein (Fig. 4C). Together, these observations suggested that the IFN-α treatment of WT276 cells increases ERα mRNA levels primarily through a transcriptional mechanism and protein synthesis is required for a rapid turnover of the ERα mRNA in WT276 cells.

### Expression of the Esr1 gene is STAT1-dependent

**Figure 5:**
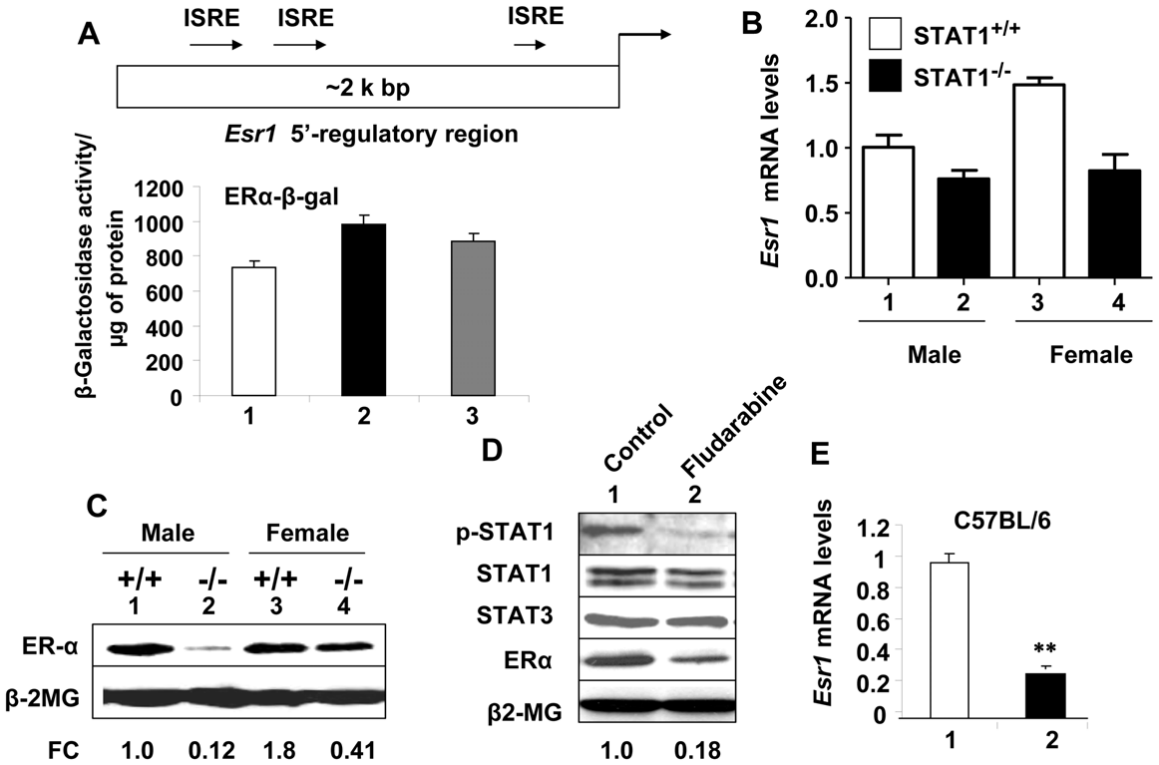
Expression of the *Esr1* gene is dependent on activation of STAT1. (A) Top panel: Schematic presentation of the 5′-regulatory region of the murine *Esr1* gene (the NCBI accession # for the sequence: NT_039490.7) and potential *cis*-elements that are predicted to render the gene responsive to the IFN treatment. The regulatory sequence for the gene is derived from the C57BL/6J strain of mice. The regulatory region includes three potential ISREs for binding of activated STAT1 (ISGF3) transcription factor. Bottom panel: Sub-confluent cultures of WT276 cells in a 6-well plate were transfected with ERα promoter-β-galactosidase plasmid (2 µg) along with pRL-TK (0.2 µg) plasmid using the FuGene 6 transfection agent. 24 h after transfections, cells were either left untreated treated (column1), treated with IFN-α (column 2), or IFN-γ (column 3). 40–45 h after transfections, cells were lysed and the lysates were processed for estimation of protein followed by β-galactosidase activity assays. (B) Total RNA was isolated from wilt-type (columns 1 and 3) or STAT1-null (columns 2 and 4) total splenic cells that were prepared from male or age-matched female (age ∼8 weeks) mice. The RNA was analyzed by quantitative real-time PCR for steady-sate levels of *Esr1* mRNA. (C) Total cell extracts were prepared from wilt-type (lanes 1 and 3) or STAT1-null (lanes 2 and 4) total splenic cells that were prepared from male or age-matched female (age ∼8 weeks) mice. The RNA was analyzed by quantitative real-time PCR for steady-sate levels of *Esr1* mRNA. Fold change in ERα protein levels is indicated below the Figure. (D) Total cell isolated from C57BL/6 were either left untreated (lane 1) or treated with fludarabine (lane 2) for 24 h. Total cell extracts were prepared and analyzed by immunoblotting using antibodies specific to the indicated proteins. Fold change in ERα protein levels is indicated below the Figure. (E) Total cell isolated from C57BL/6 were either left untreated (lane 1) or treated with fludarabine (lane 2) for 24 h. Total RNA was prepared and steady-state levels of Esr1 mRNA were analyzed by quantitative real-time PCR. Results are mean values of triplicate experiments and error bars represent standard deviation (^**^ *p*<0.005).

Transcription-dependent increase in ERα mRNA levels in IFN-treated cells (Fig. 4A) and an increased activating phosphorylation of STAT1 in IFN-α treated B cells, which associated with increased expression of ERα (Fig. 2E), prompted us to determine whether the IFN-treatment indeed activates the transcriptional of the *Esr1* gene. As shown in Fig. 5A (Top panel), consistent with the presence of three potential interferon-sensitive response elements (ISREs) consensus sequence (TTCCCGGAA) in the 5′-regulatory region of the *Esr1* gene, treatment of WT276 cells with IFN-α stimulated the activity of a reporter gene, the transcription of which was driven by the 5′-reglatory region of the murine *Esr1* gene [32]. Interestingly, consistent with our earlier observations (Fig. 1A) the stimulation of the reporter activity was relatively more in the IFN-α treated cells than IFN-γ. To further investigate how IFN-signaling activates the transcription of the *Esr1* gene, we compared basal steady-sate levels of ERα mRNA and protein between wild type and STAT1-null male and female splenocytes. As shown in Fig. 5, steady-state levels of ERα mRNA (Fig. 5B) and protein (Fig. 5C) were significantly lower in STAT1-null male and females as compared to the wild-type age-matched mice. Consistent with a role for STAT1 in IFN-mediated signaling in transcriptional activation of the *Esr1* gene, we noted that treatment of C57BL/6 splenocytes with fludarabine, an inhibitor of STAT1 phosphorylation [36], which resulted in inhibition of STAT1 phosphorylation (Fig. 5D), was associated with significantly reduced levels of ERα protein (Fig. 5D) and mRNA (Fig. 5E). Moreover, treatment of cells with JNK inhibitor II (SP600125, 60 nM in DMSO) did not result in any measurable decreases in the ERα protein levels (data not shown). Thus, ruling out IFN-mediate regulation of *Esr1*expression through the JNK/AP-1 pathway.

Our observations that basal levels of pSTAT1 are relatively higher in (NZB × NZW) F_1_ females than the age-matched males (Fig. 2E) and STAT1-null mice express relatively low levels of ERα (Fig. 5C) prompted us to compare the specific DNA-binding activities of STAT1 between males and female B cells. This approach revealed that the specific DNA-binding activity of STAT1 in nuclear extracts from C57BL/6 female B cells was measurably higher than the age-matched males in gel-mobility shift assays (data not shown). Moreover, IFN-treatment of cells increased the DNA-binding relatively in extracts from both female and male mice; however, the increase in the DNA-binding was higher in extracts from females than males. Together, these observations demonstrated that IFN-signaling up-regulates the expression of the murine *Esr1* gene in gender-dependent manner through the activation of STAT1.

### The IFN and E2-signaling Cooperate to Activate Transcription

**Figure 6:**
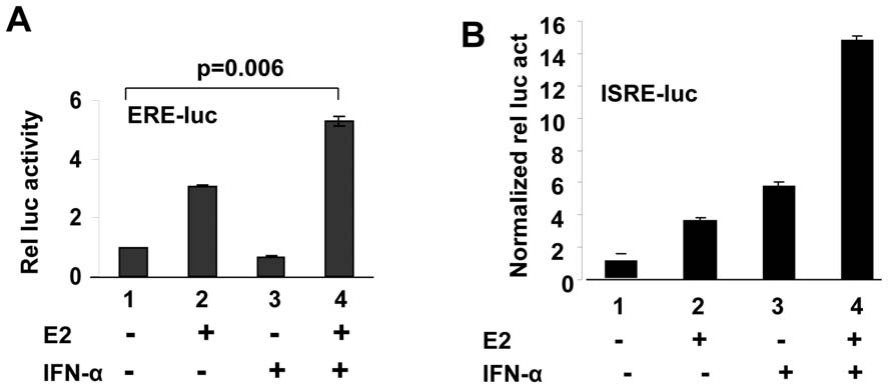
The IFN and E2-signaling cooperate to activate transcription of genes. (A) Sub-confluent cultures of WT276 cells in a 6-well plate were transfected with ERE-luc-reporter plasmid (2 µg) along with pRL-TK (0.2 µg) plasmid using FuGENE 6 transfection reagent. 24 h after transfections, cells were either left untreated (column 1), treated with E2 (column 2), IFN-α (column 3) or E2 and IFN-α (column 4). 40–45 h after transfections, cells were processed for dual luciferase activity. Result are mean values of triplicate experiments and error bars represent standard deviation (p value is 0.006). (B) Sub-confluent cultures of WT276 cells in a 6-well plate were transfected with ISRE-luc-reporter plasmid (2 µg) along with pRL-TK (0.2 µg) plasmid using FuGENE 6 transfection reagent. 24 h after transfections, cells were either left untreated (column 1), treated with E2 (column 2), IFN-α (column 3) or E2 and IFN-α (column 4). 40–45 h after transfections, cells were processed for dual luciferase activity.

Up-regulation of ERα expression by IFN-signaling in the murine cells in the above experiments prompted us to determine whether the IFN and E2-signaling cooperate with each other to activate transcriptional of target genes. As shown in Fig. 6A, treatment of WT276 cells with E2 stimulated the activity of an E2-responsive reporter about 3-fold (compare column 2 with 1). However, treatment of cells with IFN-α (in the absence of E2) did not result in stimulation of the activity of the reporter (compare column 3 with 1). Interestingly, treatment of cells with both E2 and IFN-α resulted in significantly increased stimulation of the activity of reporter (compare column 4 with 2). Because treatment of WT276 cells with E2 did not result in measurable increases (or decreases) in the ERα mRNA levels (data not shown), the above observations indicated that the IFN-induced levels of ERα increase the E2-mediated transcription of the ERα target genes.

Because E2 treatment of ER-positive cells is known to result in production of IFN-γ in a variety of cells [17]–[19], which up-regulates expression of IRF9 (a component of the ISGF3 transcription factor; ref. 29), we also tested whether treatment of cells with E2 alone or both E2 and IFN-α has any effect on expression of an IFN-responsive reporter gene. As shown in Fig. 6B, treatment of WT276 cells with E2 alone resulted in ∼3-fold stimulation of the activity of the ISRE-luc-reporter, an IFN-responsive reporter. Furthermore, treatment of cells with IFN-α alone stimulated the activity of the reporter ∼5-fold. Interestingly, treatment of cells with both IFN-α and E2 stimulated the activity of reporter ∼14-fold. Together, our observations indicated that both E2 and IFN-α signaling cooperate with each other to activate the transcription of certain ERα and IFN-responsive genes.

### Sex Bias in the Expression of IFN and E2-responsive Genes

**Figure 7:**
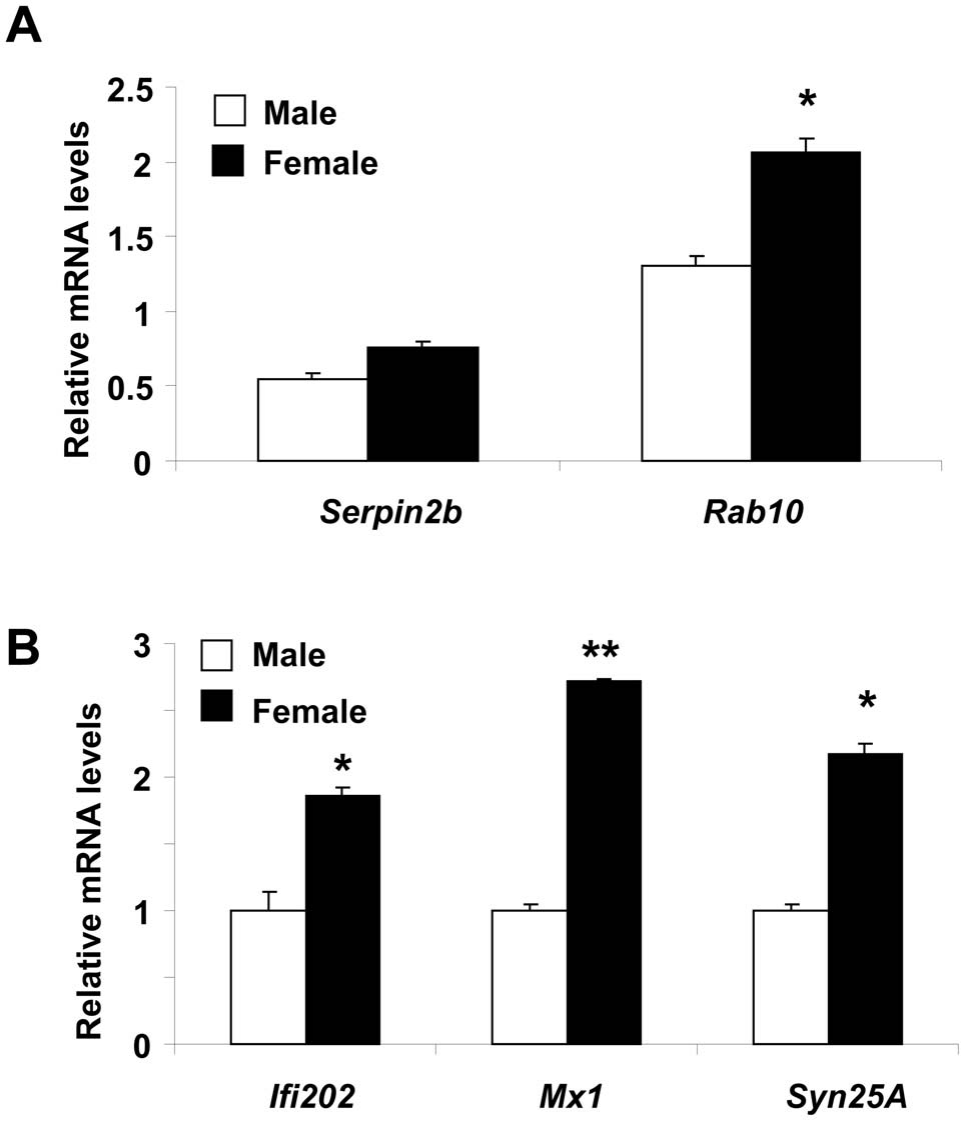
Sex bias in the expression of IFN and E2-responsive genes. (A and B) Total RNA isolated from pre-autoimmune (age ∼8 weeks) male or age-matched female (NZB × NZW) F_1_ mice was analyzed for steady-state levels of the indicated known estrogen-responsive (A) and IFN-responsive (B) genes by quantitative real-time PCR. Results are mean values of triplicate experiments and error bars represent standard deviation (^*^ *p*<0.05; ^**^ *p*<0.005).

Increased steady-state levels of ERα mRNA (Fig. 2A) protein (Fig. 2B and C) in splenic cells from (NZB × NZW) F_1_ female mice as compared to age-matched male mice and cooperation between the IFN and E2-signaling in cells to activate transcription of reporter genes (Fig. 6) prompted us to investigate whether the expression of E2 or IFN-responsive genes is differentially regulated between male and female (NZB × NZW) F_1_ lupus-prone mice. As shown in Fig. 7A, we noted that steady-state levels of mRNAs encoded by two E2-responsive genes were relatively higher in female (NZB × NZW) F_1_ mice than the age-matched males (Fig. 7A). Similarly, steady-state levels of mRNA encoded by three IFN-responsive genes were relatively higher in female mice than the age-matched male mice (Fig. 7B). Together, these observations demonstrated a sex bias in the expression of both E2 and IFN-responsive genes.

## Discussion

The development of SLE is known to have a strong sex bias [3]–[6]. Moreover, peak SLE disease incidence in women occurs during the early reproductive years (ages 20–30 years) [37]. Additionally, risk of SLE development is associated with the use of combined oral contraceptives [38]. Studies have revealed that PBMCs from SLE patients overexpress IFN-α-inducible genes as compared with healthy individuals [7] and high serum IFN-α level is a heritable risk factor for SLE development [37]. Notably, activation of TLR7-induced signaling is associated with higher IFN-α production in females [39] and the peak time frame for lupus onset in women coincides with an increase in IFN-α activity [37]. In light of the above observations, our observations that: (i) activation of IFN-signaling up-regulates the expression of ERα (Figs. 1, 2 and 3); and (ii) E2 and IFN-signaling cooperate to activate transcription of certain target genes (Figs. 6 and 7) provide support for the idea that the female sex hormone estrogen and increased levels of IFN-α contribute to sex bias in SLE through the activation of a mutually positive feedback loop.

A recent study [40] has revealed that estrogen treatment of splenocytes enhances STAT1 DNA-binding activity without increasing the levels of phosphorylated and total STAT1. Furthermore, the study also noted that estrogen induces serine protease-mediated proteolysis of STAT1, which may alter and enhance the activity of the transcription factor. In contrast to this report, we noted that steady-state levels of phospho-STAT1 and total STAT1 were consistently higher in splenocytes from female mice than the age-matched males. Moreover, we did not detect any additional forms of the STAT1 in extracts from female mice as compared to males (data not shown). Therefore, further work will be needed to resolve this apparent discrepancy.

**Figure 8:**
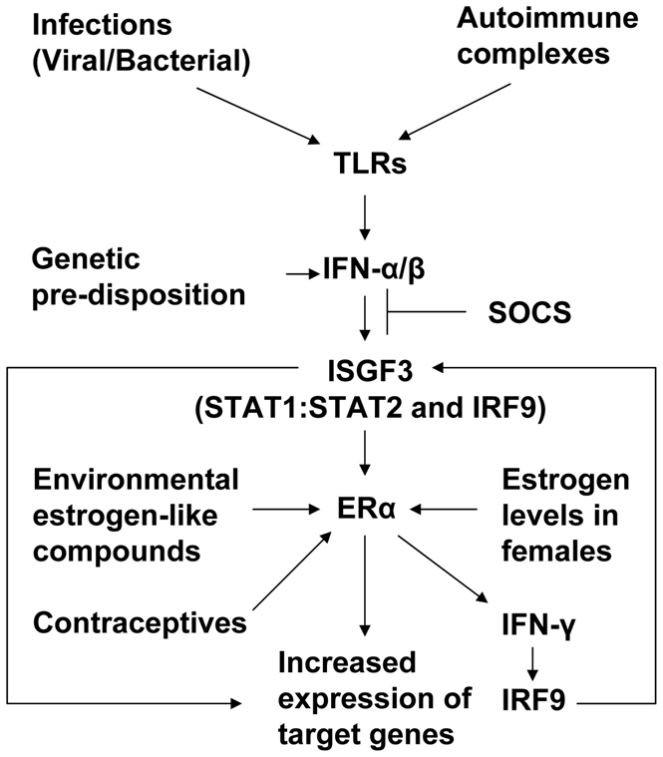
Cooperation between the IFN and E2-signaling in sex bias in SLE in mice. Increased levels of type I IFNs up-regulate expression of ERα. Activation of ERα by the female sex hormone estrogen leads to up-regulation of IFN-γ and IFN-γ-inducible IRF9. Increased levels of the IRF9 potentiate ISGF3-mediated transcription of IFN-inducible genes, which mediate the immunomodulatory functions of the IFNs.

A study [10] revealed that treatment of BALB/c mice with ER-subtype-selective agonists that results in activation of ERα, but not ERβ, plays a major role in estrogen-induced thymic atrophy and thymic T cell and splenic B cell phenotype alterations. Moreover, the study also revealed that ERα, but not ERβ, mediates the estrogen-induced up-regulation of IFN-γ. Similarly, a recent study has demonstrated a role for ERα in E2-induced development of the lupus phenotype in mice [16]. Consistent with these studies, generation of ERα knockout (NZB × NZW)F_1_ mice and their characterization revealed that E2 through ERα promotes lupus disease, in part, by inducing the IFN-γ production [15]. Moreover, estrogen is known to enhance IFN-γ production by CD11c^+^ cells [18]. Together, these observations raise the possibility that estrogen signaling through ERα in certain strains of female mice up-regulates expression of IFN-inducible genes, in part, by increasing the production of IFN-γ (Fig. 8).

The murine *Esr1* gene is transcribed from a complex transcription unit with multiple potential promoters and upstream regulatory sequences [32], [34]. Consistent with this observation, multiple transcription start sites have been identified in the regulatory region of the gene. Moreover, the promoter of the *Esr1* gene is reported to be relatively weak [33]. Therefore, our observations that treatment of cells with IFN-α or IFN-γ resulted in a modest stimulation of the activity of the reporter, the expression of which was driven by the 5′-regulatory region (∼5-kb) of the *Esr1* gene (Fig. 5A), are consistent with the above reports.

A study [26] has noted differences in estrogen receptors levels between BALB/c mice, which do not get autoimmune disease and two strains that do (MRL/MP-*lpr*/*lpr* and NZB/W mice). Therefore, our observations that basal as well as IFN-induced levels of ERα were relatively higher in non lupus-prone (C57BL/6) as well as lupus-prone (NZB × NZW) F_1_ female mice as compared to the age and strain-matched males will require further work to determine whether other factors, such as promoter polymorphisms in the *Esr1* gene, also contribute to differential expression of ERα in certain strains of mice.

Notably, a study has provided evidence that the XX sex chromosome complement, as compared with XY, confers greater susceptibility to certain autoimmune diseases, such as experimental autoimmune encephalomyelitis (EAE) and pristane-induced lupus [41]. However, it remains unclear whether the XX sex chromosome complement also contributes to sex bias in mouse models of lupus disease, such as (NZB × NZW) F_1_, which spontaneously develop the disease. Therefore, further work will be needed to investigate the role of XX sex chromosomes in these mouse models of the disease.

In summary, our observations provide support for our model (Fig. 8). The model predicts that increased levels of IFNs (IFN-α or IFN-γ) in serum of SLE patients and certain lupus-prone strains of female mice, by up-regulating the expression of ERα, potentiate the expression of certain E2 and IFN-responsive genes. Notably, increased expression of the IFN-inducible genes is associated with the active disease in SLE patients [20], [24] and certain lupus-prone strains of mice [26]. Importantly, increased expression of these IFN-inducible genes is associated with increased survival of autoreactive immune cells and autoimmunity [20], [24], [31]. Therefore, our observations concerning a mutually positive feedback loop between IFNs and ERα in mice provide a potential molecular basis for the sex bias in SLE. Further work will be needed to determine whether increased levels of type I IFN in SLE patients are associated with up-regulation of ERα expression and active SLE.
